# Quercetin, Inflammation and Immunity

**DOI:** 10.3390/nu8030167

**Published:** 2016-03-15

**Authors:** Yao Li, Jiaying Yao, Chunyan Han, Jiaxin Yang, Maria Tabassum Chaudhry, Shengnan Wang, Hongnan Liu, Yulong Yin

**Affiliations:** 1 Institute of Animal Nutrition, Northeast Agricultural University, Harbin 150030, China; qiumengyouzhi@163.com (J.Y.); hanchunyan920317@163.com (C.H.); jiaxinyang8040@163.com (J.Y.); mariach046@yahoo.com (M.T.C.); 13674620839@163.com (S.W.); 2 Scientific Observing and Experimental Station of Animal Nutrition and Feed Science in South-Central China, Ministry of Agriculture, Hunan Provincial Engineering Research Center of Healthy, Livestock, Key Laboratory of Agro-ecological Processes in Subtropical Region, Institute of Subtropical, Agriculture, Chinese Academy of Sciences, Changsha 410125, China

**Keywords:** quercetin, inflammation, immune function, dietary sources, metabolism

## Abstract

*In vitro* and some animal models have shown that quercetin, a polyphenol derived from plants, has a wide range of biological actions including anti-carcinogenic, anti-inflammatory and antiviral activities; as well as attenuating lipid peroxidation, platelet aggregation and capillary permeability. This review focuses on the physicochemical properties, dietary sources, absorption, bioavailability and metabolism of quercetin, especially main effects of quercetin on inflammation and immune function. According to the results obtained both *in vitro* and *in vivo*, good perspectives have been opened for quercetin. Nevertheless, further studies are needed to better characterize the mechanisms of action underlying the beneficial effects of quercetin on inflammation and immunity.

## 1. Introduction

Quercetin, a flavonoid found in fruits and vegetables, has unique biological properties that may improve mental/physical performance and reduce infection risk [1]. These properties form the basis for potential benefits to overall health and disease resistance, including anti-carcinogenic, anti-inflammatory, antiviral, antioxidant, and psychostimulant activities, as well as the ability to inhibit lipid peroxidation, platelet aggregation and capillary permeability, and to stimulate mitochondrial biogenesis [2]. Therefore, there is a pressing need for well-designed clinical trials to evaluate this novel dietary supplement further. This article reviews effects of quercetin on inflammation and immunity in mental and physical performance and health.

## 2. Physicochemical Properties of Quercetin

**Figure 1:**
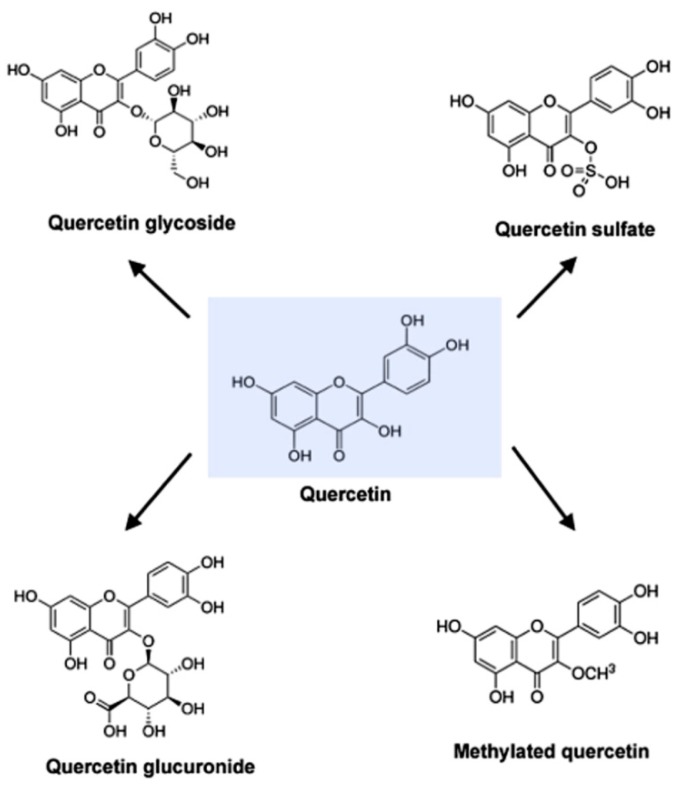
Molecular structure of quercetin, quercetin glycoside, quercetin glucuronide, quercetin sulfate and methylated quercetin.

Quercetin is categorized as a flavonol, one of the six subclasses of flavonoid compounds. The name has been used since 1857, and is derived from *quercetum* (oak forest), after *Quercus*. It is a naturally occurring polar auxin transport inhibitor [3]. The International Union of Pure and Applied Chemistry (IUPAC) nomenclature for quercetin is 3, 3′, 4′, 5, 7-pentahydroxyflvanone (or its synonym 3, 3′, 4′, 5, 7-pentahydroxy-2-phenylchromen-4-one). This means that quercetin has an OH group attached at positions 3, 5, 7, 3′, and 4′. Common forms of quercetin were shown in Figure 1.

Quercetin (C15H10O7) is an aglycone, lacking an attached sugar. It is a brilliant citron yellow needle crystal and entirely insoluble in cold water, poorly soluble in hot water, but quite soluble in alcohol and lipids. A quercetin glycoside is formed by attaching a glycosyl group (a sugar such as glucose, rhamnose, or rutinose) as a replacement for one of the OH groups (commonly at position 3). The attached glycosyl group can change the solubility, absorption, and *in vivo* effects. As a general rule of thumb, the presence of a glycosyl group (quercetin glycoside) results in increased water solubility compared to quercetin aglycone [4,5].

A quercetin glycoside is unique by the attached glycosyl group. Generally, the term quercetin should be used to describe the aglycone only; however, the name is occasionally used to refer to quercetin-type molecules, including its glycosides in research and the supplement industry.

## 3. Dietary Sources of Quercetin

Quercetin-type flavonols (primarily as quercetin glycosides), the most abundant of the flavonoid molecules, are widely distributed in plants. They are found in a variety of foods including apples, berries, Brassica vegetables, capers, grapes, onions, shallots, tea, and tomatoes, as well as many seeds, nuts, flowers, barks, and leaves. Quercetin is also found in medicinal botanicals, including *Ginkgo biloba*, *Hypericum perforatum*, and *Sambucus canadensis* [6,7,8]. In red onions, higher concentrations of quercetin occur in the outermost rings and in the part closest to the root, the latter being the part of the plant with the highest concentration [9]. One study found that organically grown tomatoes had 79% more quercetin than chemically grown fruit [10]. Quercetin is present in various kinds of honey from different plant sources [11]. Food-based sources of quercetin include vegetables, fruits, berries, nuts, beverages and other products of plant origin [12]. In the determined food, the highest concentration is 234 mg/100 g of edible portion in capers (raw), the lowest concentration is 2 mg/100 g of edible portion in black or green tea (*Camellia sinensis*) [13].

Dietary intake of quercetin was different in several countries. The estimated flavonoid intake ranges from 50 to 800 mg/day (quercetin accounts for 75%), mostly depending on the consumption of fruits and vegetables and the intake of tea [14]. In the Suihua area of northern China, quercetin intake was reported to be 4.37 mg/day, where the main food sources of flavonol was apples (7.4%), followed by potatoes (3.9%), lettuce (3.8%) and oranges (3.8%) [15], whereas the average quercetin intake was 4.43 mg/day, with apple (3.7%), potato (2.5%), celery (2.2%), eggplant (2.2%), and actinidia (1.6%) being the main food sources of quercetin in Harbin, China [16]. The most recent study showed that quercetin intake is about 18 mg/day for Chinese healthy young males. In the USA, flavonol intake is about 13 mg/day for U.S. adults, while quercetin represents three-quarters of this amount. The mean quercetin intake was approximately 14.90 to 16.39 mg per day. Onions, tea, and apples contained high amounts of quercetin [17]. In Japan, the average and median quercetin intakes were 16.2 and 15.5 mg/day, respectively; the quercetin intake by men was lower than that by women; and the quercetin intakes showed a low correlation with age in both men and women. The estimated quercetin intake was similar during summer and winter. Quercetin was mainly ingested from onions and green tea, both in summer and in winter. Vegetables, such as asparagus, green pepper, tomatoes, and red leaf lettuce, were good sources of quercetin in summer [18]. In Australia, black and green teas were the dominant sources of quercetin. Other sources included onion, broccoli, apple, grape, and beans [19]. Analysis of the 24-h recall data indicated an average adult intake of total flavonoids (>18 years) of 454 mg/day. Apple was the most important source of quercetin until age 16–18 years, after which onion became an increasingly important prominent source [19]. In Spain, the average daily intake of quercetin is 18.48 mg/day, which is significantly higher than that in the United States (9.75 mg/day), based on sources like tea, citrus fruits and juice, beers and ales, wines, melon, apples, onions, berries and bananas [20].

## 4. Absorption, Bioavailability and Metabolism of Quercetin

The first investigation on the pharmacokinetics of quercetin in humans suggested very poor oral bioavailability after a single oral dose (~2%). The estimated absorption of quercetin glucoside, the naturally occurring form of quercetin, ranges from 3% to 17% in healthy individuals receiving 100 mg. The relatively low bioavailability of quercetin may be attributed to its low absorption, extensive metabolism and/or rapid elimination.

### 4.1. Absorption

Quercetin glycosides might be differently absorbed based on the type of sugar attached [21]. Available evidence indicates that quercetin glucosides (like those found predominantly in onion or shallot flesh) are far better absorbed than its rutinosides (the major quercetin glycoside in tea). The glucosides are efficiently hydrolyzed in the small intestine by beta-glucosidases to the aglycone form, much of which is then absorbed [22]. Quercetin glucuronic acid and its sulfuric acid derivatives were more easily absorbed than quercetin [22]. Thereafter, its absorption is affected by differences in its glycosylation, the food matrix from which it is consumed, and the co-administration of dietary components such as fiber and fat [23]. Thus different sugar types and sugar group conjugation sites will result in absorption variation.

Quercetin and derivatives are stable in gastric acid; however, there were no reports whether they can be absorbed in stomach. Studies suggest that quercetin is absorbed in the upper segment of small intestinal [24,25].

Among quercetin’s derivatives, conjugated forms of its glycosides are better absorbed than quercetin. Purified quercetin glucosides are capable of interacting with the sodium dependent glucose transport receptors in the mucosal epithelium and may therefore be absorbed by the small intestine *in vivo* [21].

### 4.2. Transformation and Transportation

After absorption, quercetin becomes metabolized in various organs including the small intestine, colon, liver and kidney. Metabolites formed in the small intestine and liver by biotransformation enzymes include the methylated, sulfo-substituted and glucuronidated forms [26,27]. A study regarding the tissue distribution in rats and pigs has shown that the highest accumulation of quercetin and its metabolites are found in (rat) lung and (pig) liver and kidney [28].

Quercetin and derivatives are transformed into various metabolites (phenolic acid) by enteric bacteria and enzymes in intestinal mucosal epithelial cells. These metabolites are absorbed, transformed or excreted later. Moreover, bacteria ring fission of the aglycon occurs in both the small intestine and colon, resulting in the breakdown of the backbone structure of quercetin and the subsequent formation of smaller phenolics [29].

Quercetin metabolites analyzed in plasma and liver samples have shown that the concentrations of its derivatives in the liver were lower than those in plasma, and the hepatic metabolites were intensively methylated (90%–95%) [30]. Limited studies suggest that quercetin was methylated, vulcanized and glucuronidated in liver [31].

Continuous intake of diet containing quercetin accumulated in blood and significantly increased quercetin concentration in plasma, which was significantly correlated to its dietary content [32]. Quercetin is present in a conjugated form in human blood whose major form is glycoside [33]. While isorhamnetin and glucoside acid-sulfated derivatives of quercetin account for 91.5% of its metabolites, other metabolites include its glucuronoside and methylated form [34]. Boulton also found that quercetin conjugated plasma protein (albumin account for 99.4%), thus decreased its bioavailability in cells [35].

### 4.3. Excretion

The limited research suggests that quercetin and its metabolites tend to accumulate in the organs involved in its metabolism and excretion, and that perhaps mitochondria might be an area of quercetin concentration within cells [36,37,38,39,40,41,42]. Kidney is a major organ of excretion. Quercetin concentration in urine increased with the increasing dose and time after intake of fruit juice was ingested in human [36], perhaps benzoic acid derivatives are common metabolite of quercetin [37]. Human subjects can absorb significant amounts of quercetin from food or supplements, and elimination is quite slow, with a reported half-life ranging from 11 to 28 h [38]. The average terminal half-life of quercetin is 3.5 h [39]. The total recovery of C-quercetin in urine, feces and exhaled air is highly variable, depending on the individual [40]. A high amount of absorbed quercetin is extensively metabolized and eventually eliminated by the lungs [41]. Additional literature suggests that isoquercetin (glycosylated quercetin) is more completely absorbed than quercetin in the aglycone form, and that the simultaneous ingestion of quercetin with vitamin C, folate and additional flavonoids improves bioavailability [38,42].

All of these studies indicate that quercetin glucosides is absorbed in the upper segment of small intestinal, then is methylated, sulfo-substituted and glucuronidated by biotransformation enzymes in the small intestine and liver, and is finally excreted by kidney in urine.

## 5. Effect of Quercetin on Inflammation and Immune Function

### 5.1. In Vitro

#### 5.1.1. Anti-Inflammation and Promotion of Immunity

Quercetin was reported as a long lasting anti-inflammatory substance that possesses strong anti-inflammatory capacities [43,44]. It possesses anti-inflammatory potential that can be expressed on different cell types, both in animal and human models [45,46,47,48,49,50,51,52,53]. It is known to possess both mast cell stabilizing and gastrointestinal cytoprotective activity [54]. It can also play a modulating, biphasic and regulatory action on inflammation and immunity [53]. Additionally, quercetin has an immunosuppressive effect on dendritic cells function [55].

#### 5.1.2. Mechanism of Action

Several studies *in vitro* using different cell lines have shown that quercetin inhibits lipopolysaccharide (LPS)-induced tumor necrosis factor α (TNF-α) production in macrophages [45] and LPS-induced IL-8 production in lung A549 cells [46]. Moreover, in glial cells it was even shown that quercetin can inhibit LPS-induced mRNA levels of TNF-α and interleukin (IL)-1α, this effect of quercetin resulted in a diminished apoptotic neuronal cell death induced by microglial activation [47]. Quercetin inhibits production of inflammation-producing enzymes (cyclooxygenase (COX) and lipoxygenase (LOX)) [48,49]. It limits LPS-induced inflammation via inhibition of Src- and Syk-mediated phosphatidylinositol-3-Kinase (PI3K)-(p85) tyrosine phosphorylation and subsequent Toll Like Receptor 4 (TLR4)/MyD88/PI3K complex formation that limits activation of downstream signaling pathways in RAW 264.7 cells [50]. It can also inhibit FcεRI-mediated release of pro-inflammatory cytokines, tryptase and histamine from human umbilical cord blood-derived cultured mast cells (hCBMCs); this inhibition appears to involve in inhibition of calcium influx, as well as phospho-protein kinase C (PKC) [51]. The study of quercetin against H_2_O_2_-induced inflammation showed the protective effects of quercetin against inflammation in human umbilical vein endothelial cells (HUVECs) and indicated that the effect was mediated via the downregulation of vascular cell adhesion molecule 1 (VCAM-1) and CD80 expression [52].

Quercetin significantly induces the gene expression as well as the production of Th-1 derived interferon-γ (IFN-γ) and down-regulates Th-2 derived interleukin 4 (IL-4) by normal peripheral blood mononuclear cells (PBMC). Furthermore, quercetin treatment increased the phenotypic expression of IFN-γ cells and decreased IL-4 positive cells by flow cytometry analysis, which corroborate with protein secretion and gene expression studies. These results suggest that the beneficial immuno-stimulatory effects of quercetin may be mediated through the induction of Th-1 derived cytokine, IFN-γ, and inhibition of Th-2 derived cytokine, IL-4 [56].

Quercetin is able to inhibit matrix metalloproteinases, which are normally inhibited by plasminogen activator inhibitor 1 (PAI-1) in human dermal fibroblasts [57]. IL-1-stimulated IL-6 production from human mast cells is regulated by biochemical pathways distinct from IgE-induced degranulation, and quercetin can block both IL-6 secretion and two key signal transduction steps involved [58].

Quercetin is known to possess both mast cell stabilizing and gastrointestinal cytoprotective activity. A study demonstrates that quercetin has a direct regulatory effect on basic functional properties of immune cells which may be mediated by the extracellular regulated kinase 2 (Erk2) mitogen-activated protein (MAP) kinase signal pathway in human mitogen-activated PBMC and purified T lymphocytes [54].

The property proves inhibitory to a huge panoply of molecular targets in the micromolar concentration range, either by down-regulating or suppressing many inflammatory pathways and functions. Quercetin has shown a biphasic behavior in basophils at nanomolar doses and hence its action on cells involved in allergic inflammation. Quercetin affects immunity and inflammation by acting mainly on leukocytes and targeting many intracellular signaling kinases and phosphatases, enzymes and membrane proteins are often crucial for a cellular specific function. However, the wide group of intracellular targets and the elevated number of natural compounds potentially effective as anti-inflammatory therapeutic agents, asks for further insights and evidence to comprehend the role of these substances in animal cell biology [53].

*In vitro* treatment of activated T cells with quercetin blocks IL-12-induced tyrosine phosphorylation of JAK2, TYK2, STAT3, and STAT4, resulting in a decrease in IL-12-induced T cell proliferation and Th1 differentiation [59].

**Figure 2:**
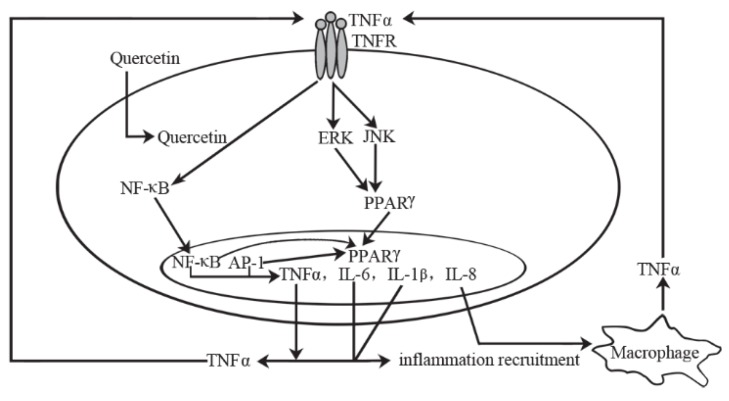
Working model on how quercetin block tumor necrosis factor-α (TNFα)-mediated inflammation. Quercetin prevents TNF-α from directly activating extracellular signal-related kinase (ERK), c-Jun NH_2_-terminal kinase (JNK), c-Jun, and nuclear factor-κB (NF-κB), which are potent inducers of inflammatory gene expression and protein secretion. In addition, quercetin may indirectly prevent inflammation by increasing peroxisome proliferator-activated receptor c (PPARγ) activity, thereby antagonizing NF-κB or activator protein-1(AP-1) transcriptional activation of inflammatory genes. Together, these block TNF-α-mediated induction of inflammatory cascades.

Taken as *in vitro* together, the possible pathway of quercetin on inflammation and immune function is as follows (Figure 2).

The main action of quercetin on inflammation and immune function *in vitro* is summarized in the Table 1.

### 5.2. In Vivo

#### 5.2.1. Animal Models

Quercetin exerts inflammation and immune modulating activity in several murine models of autoimmunity. *In vivo*, animal experiments also support an anti-inflammatory effect. Quercetin ameliorates the inflammatory response induced by carrageenan [60] and a high-fat diet [61]. Quercetin reduced visceral adipose tissue TNF-α and nitric oxide production and downregulated nitric oxide synthase (NOS) expression in obese Zucker rats [62]. In chronic rat adjuvant induced arthritis, quercetin decreased clinical signs of arthritis compared to untreated controls [63].

In rats, post-trauma administration of quercetin improves recovery of motor function after acute traumatic spinal cord injury. Intraperitoneal (IP) doses of 5–100 micromoles quercetin/kg body weight resulted in half or more of the animals walking, although with deficit [64]. This ability to promote recovery from spinal cord injury appears to be highly dependent on the dose and frequency of dosing. In this study a lower IP dose was ineffective. In another study, compared to an untreated control group of animals (none of which recovered motor function sufficient to walk), quercetin administration twice daily for three or 10 days resulted in about 50 percent of the animals recovering sufficient motor function to walk. However, when quercetin was injected three times daily, none of the nine animals recovered the ability to walk [65].

#### 5.2.2. Mechanism of Action in Animal

Study has shown that quercetin exerted protective effect against irradiation-induced inflammation in mice through increasing cytokine secretion [66]. Quercetin possesses activity against isoproterenol-induced myocardial oxidative injury and immunity function impairment, and that the mechanism of pharmacological action was related at least in part to the antioxidant activity of quercetin [67]. Quercetin decreased histological signs of acute inflammation in the treated animals in a dose-dependent manner via suppressing leucocyte recruitment, decreasing chemokine levels and levels of the lipid peroxidation end-product malondialdehyde, and increasing antioxidant enzyme activity in experimental rat model [68].

Quercetin ameliorated experimental allergic encephalomyelitis (EAE) by blocking IL-12 signaling and Th1 differentiation [58] and experimental autoimmune myocarditis (EAM) in Dark Agouti rats by interfering with production of pro-inflammatory (TNF-α and IL-17) and/or anti-inflammatory (IL-10) cytokines [69]. Quercetin most likely universally suppresses the accumulation and activation of immune cells, including anti-inflammatory cells, whereas it specifically increased gene expression associated with mitochondrial oxidative phosphorylation in Western diet-induced obese mice. Suppression of oxidative stress and NF-κB activity likely contributed to the prevention of the accumulation and activation of immune cells and resulting chronic inflammation of epididymal adipose tissue in Western diet-induced obese mice [70].

#### 5.2.3. Clinical Studies

Diet supplementation with combinations of resveratrol, pterostilbene, morin hydrate, quercetin, δ-tocotrienol, riboflavin, and nicotinic acid reduces cardiovascular risk factors in humans when used as nutritional supplements with, or without, other dietary changes in healthy seniors and hypercholesterolemic subjects [71].

In a randomized, double-blinded, placebo-controlled trial, 1002 subjects took 500 or 1000 mg/day quercetin or a placebo for 12 weeks. For the group as a whole, quercetin supplementation had no significant influence on rates of upper respiratory tract infections (URTI) compared to placebo. In a subgroup of subjects age 40 or older who self-rated themselves as physically fit, 1000 mg/day quercetin resulted in a statistically significant reduction in total sick days and symptom severity associated with URTI [72]. Female subjects were supplemented with 500 or 1000 mg/day quercetin or placebo for 12 weeks. While quercetin supplementation significantly increased plasma quercetin levels, it had no influence on measure of immune function [73]. Quercetin (100 mg/day) did not alter exercise-induced changes in several measures of immune function following three days of intense exercise in trained athletes, but it significantly reduced URTI incidence (1 of 20 subjects in active *versus* 9 of 20 in placebo group) during the two-week post-exercise period [74]. A similar lack of effect on strenuous exercise-induced immune system perturbation was found in subjects who took 1000 mg/day of quercetin for three weeks before, during, and continuing for two weeks after the 160-km Western States Endurance Run. In this study, however, there were no differences in the post-race illness rates between quercetin and placebo groups [75].

There are several studies in humans investigating the correlation of quercetin and its immunomodulatory effects. Quercetin does indeed reduce illness after intensive exercise. Again, under double-blind conditions, Nieman *et al.* showed that a supplement of 1000 mg of quercetin alone three weeks before, during and two weeks after a three-day period of 3 h of cycling in the winter resulted in a markedly lower incidence of URTI in well-trained subjects in the two weeks after the intensified training, but had no effect on exercise-induced immune dysfunction, inflammation and oxidative stress [76].

The literature is supportive of the anti-pathogenic capacities of quercetin when it is cultured with target cells and a broad spectrum of pathogens including URTI-related rhinoviruses, adenoviruses and coronaviruses. The impact of the co-ingestion of two or more flavonoids increases their bioavailability and the outcomes on immunity. Nieman *et al.* determined the influence of two weeks of 1000 mg/day quercetin compared with placebo supplementation on exercise performance and skeletal muscle mitochondrial biogenesis in untrained, young adult males. It resulted in significantly reduced post-exercise measures for both inflammation and oxidative stress, with a chronic augmentation of granulocyte oxidative burst activity [77]. When taken together, quercetin showed a successful reduction in the illness rates of exercise-stressed athletes as well as a chronic augmentation of their innate immune function.

Most *in vitro* research suggests that quercetin possesses anti-inflammation and immunological improvement. However, the results from a double-blinded, placebo-controlled, randomized trial indicated that quercetin supplementation at 500 and 1000 mg/day for 12 weeks significantly increased plasma quercetin levels but had no influence on measures of innate immune function or inflammation in community-dwelling adult females [73].

The main action of quercetin on inflammation and immune function *in vivo* is summarized in the Table 2.

These results suggest that quercetin exhibited anti-inflammation and immune-enhancement *in vitro* (cells) and *in vivo* (animals), however, studies in human did not totally support these results from cells and animals. The effect, in which quercetin acts as an immune booster in humans, needs to be further verified for future broad application.

## 6. Summary

As a widespread flavonoid, quercetin is a safe and dietary supplement based on its broad range of biological effects in animal. The results of these effects are not consistent, however, and the outcomes need to be carefully evaluated, as they are dependent on the type of subject and their level of health. Taken together, we know definitively that a quercetin glycoside is much more efficient than other forms of quercetin. In the majority of the literature, we find references to the benefits of prolonged supplementation with quercetin.

The future challenge is to investigate optimal benefits of quercetin, especially to the recommendation for the protracted intake. For example, a carbohydrate drink may have a better effect than pure quercetin preparation. The research in this area continues to determine the proper outcomes, dosing regimen and adjuvants that may amplify any perceived bioactive effects of quercetin *in vivo*.

## Figures and Tables

**Table 1:** Summary of the main effects of quercetin on inflammation and immune function *in vitro*.

Dosage	Cell Lines	Effect	Mechanism	Reference
**Cells from animals**				
100 μmol/L	Pulmonary Epithelial Cell (A549)	Anti-inflammation	PARP-1 inhibition and preservation of cellular NAD1 and energy production	[46]
100 μmol/L	N9 microglial cells		Inhibition of TNFα and IL-1α; Reduce of apoptotic neuronal cell death induced by microglial activation	[47]
3 μmol/L	Gunea pig epithelial cells		Inhibition of both cyclooxygenase and lipoxygenase	[48]
15–30 μmol/L	Rat liver epithelial (RLE) cells		Inhibition of arsenite-induced COX-2 expression mainly by blocking the activation of the PI3K signaling pathway	[49]
-	RAW 264.7 cells		Inhibition of Src- and Syk-mediated PI3K-(p85) tyrosine phosphorylation and subsequent TLR4/MyD88/PI3K complex formation that limits activation of downstream signaling pathways	[50]
**Cells from human**				
10 μmol/L	Human umbilical cord blood-derived cultured mast cells (hCBMCs)	Anti-allergic and anti-inflammation; Protective effects against cell injury; Gastrointestinal cytoprotective action	Inhibition of intracellular calcium influx and PKC theta signaling	[51]
50 or 100 µg	T lymphocyte		Blockage of interleukin-12 signaling through JAK-STAT pathway	[52]
-	Mast cell		Stabilization of mast cell and gastrointestinal cytoprotection via lactone stimulating mucus production, and inhibiting histamine and serotonin release from intestinal mast cells	
12.5–25.0 mmol/L	Human inflamed/UV-irradiated skin		Inhibition of MMP-1 and down-regulation of MMP-1 expression via an inhibition of the AP-1 activation	[54]
0–210 μmol/L	Human umbilical vein endothelial cells (HUVECs)		Downregulation of VCAM-1 and CD80 expression	[56]
0.5–50 mmol/L	Human normal peripheral blood mononuclear cells	Beneficial immuno-stimulatory effects	Induction of Th-1 derived cytokine, IFNgamma, and inhibition of Th-2 derived cytokine, IL-4	[57]
1–100 mmol/L	Human umbilical cord blood-derived cultured mast cells (hCBMCs)		Inhibition of IL-1-induced IL-6 secretion, p38 and PKC-theta phosphorylation	[58]
≥100 mmol/L or ≤50 mmol/L	Mouse endritic cells (mDCs)	Immunosuppression	Inhibition of DC activation; DC apoptosis; Downregulation of the cytokines and chemokines, disturbance of immunoregulation; Attenuation of LPS-induced DC maturation and limitation of immunostimulatory activity; downregulate of endocytosis and impairment of Ag loading; suppression of DC migration and disconnection of the induction of adaptive immune responses	[55]

**Table 2:** Summary of the main effects of quercetin on inflammation and immune function *in vivo*.

Dosage	Subjects	Effect	Mechanism	Reference
Animals				
10 mg/kg diet	Rat	Anti-inflammation	Modulation of prostanoid synthesis and cytokine production	[60]
0.8% diet	C57BL/6J mouse		Increase of energy expenditure; Decrease of interferon-γ, interleukin-1α, and interleukin-4	[61]
10 mg/kg of body weight	Zucker rat		Downregulation of visceral adipose tissue inducible nitric oxide synthase expression, increase of endothelial nitric oxide synthase expression	[62]
160 mg/kg body weight (oral administration) 60 mg/kg (intra-cutaneous injections)	Lewis rat		Inhibition of macrophage-derived cytokines and nitric oxide	[63]
10 and 40 mg/kg body weight	Mouse		Increase of cytokine (interleukin-1β and interleukin-6) secretion	[66]
5–100 micromoles /kg body weight (administered intraperitoneally) 25 µmol/kg	Wistar rat	Functional recovery of acute spinal cord injury and motor function	Decrease of secondary damage through iron chelation, No effect	[64,65]
0.05% diet	C57BL/6J mouse		Suppression of the accumulation and activation of immune cells, Suppression of oxidative stress and NFκB activity	[70]
50, 100, 150 mg/kg body weight	Wistar rat	Amelioration of immunity function impairment induced by isoproterenol; Amelioration of brain damage and neuroprotection, experimental allergic encephalomyelitis, experimental autoimmune myocarditis	Increase of activity in aspartate transaminase, creatine kinase, nitric oxide, nitric oxide synthase, interleukin-10, interleukin-1, interleukin-8 and lactate dehydrogenase	[59]
50 mg/kg	Sprague-Dawley (SD) rat		Increase of activity of endogenous antioxidant enzymes and inhibition of free radical generation	[67]
50 or 100 μg	SJL/J mice		Blockage of interleukin-12 signaling and Th1 differentiation	[68]
10 or 20 mg/kg (oral administration)	Dark Agouti rat		Interference of pro-inflammatory (TNF-α and IL-17) and/or anti-inflammatory (IL-10) cytokines production	[69]
Human				
50 and 100 mg/person	Elderly Human subject	Anti-inflammatory properties	Inhibition of proteasome (nitric oxide, C-reactive protein, γ-glutamyltransferase) activity	[71]
500 and 1000 mg/day	Human subject	Reduction of upper respiratory tract infection and total sick days; Improvement in 12-min treadmill time trial performance	No effect	[72]
1000 mg/day	Human in treadmill		No effect	[76]
500 and 1000 mg/day	Human subject	No effect on innate immune function or inflammation, illness rates	No effect	[73]
1000 mg/day	Human cyclist		No effect	[74]
1000 mg/day	Human runner		No effect	[75]
1000 mg/day	Human cyclist		No effect	[77]
